# Phytohormones and candidate genes synergistically regulate fruitlet abscission in *Areca catechu* L.

**DOI:** 10.1186/s12870-023-04562-8

**Published:** 2023-11-03

**Authors:** Jia Li, Yunche Chen, Guangzhen Zhou, Meng Li

**Affiliations:** 1 Hainan Key Laboratory for Sustainable Utilization of Tropical Bioresources, College of Tropical Crops, Hainan University, Haikou, 570228 China; 2 Coconut Research Institute, Chinese Academy of Tropical Agricultural Sciences, Wenchang, 571339 Hainan China; 3 College of Life Science and Technology, Central South University of Forestry and Technology, Changsha, Hunan 410004 P. R. China

**Keywords:** Phytohormone, Transcriptome, Fruitlet abscission, *Areca catechu* L.

## Abstract

**Background:**

The fruit population of most plants is under the control of a process named “physiological drop” to selectively abort some developing fruitlets. However, frequent fruitlet abscission severely restricts the yield of *Areca catechu*. To reveal the physiological and molecular variations in this process, we detected the variation of phytohormone levels in abscised and non-abscised fruitlets in *A. catechu*.

**Results:**

The levels of gibberellin acid, jasmonic acid, salicylic acid, abscisic acid and zeatin were elevated, while the indole-3-acetic acid and indole-3-carboxaldehyde levels were declined in the “about-to-abscise” part (AB) of abscission zone (AZ) compared to the “non-abscised” part (CK). Then the differentially expressed genes (DEGs) between AB and CK were screened based on transcriptome data. DEGs involved in phytohormone synthesis, response and transportation were identified as key genes. Genes related to cell wall biosynthesis, degradation, loosening and modification, and critical processes during fruit abscission were identified as role players. In addition, genes encoding transcription factors, such as NAC, ERF, WRKY, MADS and Zinc Finger proteins, showed differentially expressed patterns between AB and CK, were also identified as candidates.

**Conclusions:**

These results unraveled a phytohormone signaling cross talk and key genes involved in the fruitlet abscission process in *A. catechu.* This study not only provides a theoretical basis for fruitlet abscission in *A. catechu*, but also identified many candidate genes or potential molecular markers for further breeding of fruit trees.

**Supplementary Information:**

The online version contains supplementary material available at 10.1186/s12870-023-04562-8.

## Background

Organ abscission is a process under elaborate gene regulatory networks in the plant kingdom. Several regulators, including external environmental perturbations and internal signals, could lead to abscission at different developmental stages. Abscission is a complicated and highly coordinated physiological process. Organ abscission could be classified into three categories according to the causation, including senescence-driven abscission of ripe organs (fruits and seeds), metabolic abscission or physiological drop (fruitlets and non-pollinated flowers) and induced abscission (induced by high and low temperature, light intensity or pathogen) [1]. Abscission evolves the process of organ separation, which occurs in a specific position called an abscission zone (AZ). A series of physiological events take place in AZ during abscission, including abscission signals transduction, AZ cell differentiation, activation of organ separation and formation of protective layer [2, 3]. Although some key regulators have been identified in *Arabidopsis* and tomato (*Solanum lycopersicum*), more evidence, especially findings from non-model species, is needed to comprehensively understand the regulatory mechanism of organ abscission. To reveal the molecular variations during fruitlet abscission in *A. catechu*, we performed RNA-seq analysis in AZ samples at different stages during fruitlet abscission, and noticed that almost all members of the DNA binding with one finger (*DOF*) gene family showed a significant up-regulation in “about-to-abscise” AZs, indicating that *DOF* gene family plays a key role in fruitlet abscission of *A. catechu * [4].

Phytohormones play an important role in regulating the occurrence of organ abscission [5]. Auxin and ethylene were first verified to participate in abscission regulation in an antagonistic way [6]. In *Arabidopsis*, specifically reducing auxin biosynthesis in the AZ of floral organs resulted in prematurely shedding. On the contrary, disruption of auxin signaling or response in AZ delayed the floral organs shedding, suggesting that a functional auxin signaling/response pathway in AZ cells is necessary for abscission initiation [7]. Ethylene acts as a signaling molecule to induce cell separation, thus promotes abscission [8]. The *Arabidopsis* mutants, *ein2*, *ein3*, *etr1* and *ers2*, lacking an ethylene receptor or its downstream pathway members, exhibited varying degrees of delay in flower organ shedding [8]. Abscisic acid (ABA) and cytokinin are critical phytohormones regulating abscission. However, present evidence indicated that the effect of ABA and cytokinin on plant organ abscission might be exerted via auxin or ethylene rather than directly [9]. Gibberellin acid (GA) and brassinolide (BRs) are proven to be inhibitors of fruit abscission. External application of GA prevented leaf and fruit abscission in peach (*Prunus persica*) [10]. BRs inhibited ethylene-induced fruitlet abscission through the LcBZR1/2-mediated transcriptional suppression of the *LcACS1/4* and *LcACO2/3* genes in litchi (*Litchi chinensis*) [11].

On the contrary, jasmonic acid was reported as an organ abscission accelerator. External application of methyl jasmonate induced leaf abscission in soybean [12] and led to fruit abscission in apple and tomato [13, 14]. In addition, an exception was reported in tomato (*Solanum lycopersicum*) that a *SlPhyt2* gene encoding inositol hexaphosphate regulate flower abscission independently way with phytohormones. The *SlPhyt2* gene triggers the expression of an abscission-related galactosidase gene *SlTAPG4*, which can improve the abscission of tomato flowers under drought stress, and the plant xanthin PSK might also be involved in this process [15]. These evidences indicated that abscission is a complex process controlled by multiple factors.

To achieve organ separation, cell wall degradation and cell death are necessary events involved in abscission. Cellulase (CEL) and polygalacturonase (PG) are pivotal enzymes promoting cell wall degradation. The high expression of two cellulase genes (*LcCEL2* and *LcCEL8*) in litchi reduced AZ's cellulose content. Overexpression of *LcCEL2* and *LcCEL8* in *Arabidopsis* showed pronounced premature shedding in floral organs [16]. In *Arabidopsis*, a polygalacturonase gene, *PGAZAT*, whose expression is directly suppressed by the AtDof4.7 transcription factor, thereby affecting cell wall degradation and resulting in the failure of normal organ abscission [17, 18]. On the contrary, another Dof transcription factor, AtCDF4, can accelerate floral organ abscission by activating the expression of the *PGAZAT* gene [19].

Another obstacle to abscission is pectin. Pectin is essential for maintaining cell wall structure integrity, intercellular adhesion and signal transduction. In Chinese roses, pectinase was verified to participate in the shedding of stamens and petals [20], while EXPANSIN (EXP) was involved in the shedding of leaves and petals [21, 22]. PECTIN METHYLESTERASE (PME) was also reported to change the chemical composition of AZ through its hydrolysis activity, resulting in cell wall and membrane degradation [23]. In addition, a study in tomato showed that proline hydroxylation at the post-translation level also affect abscission. Silencing of the *SlP4H3* (Prolyl 4 hydroxylase 3) gene delayed the abscission of overripe tomato fruits. The down-regulation of genes encoding cell wall hydrolase (PG, CEL and EXP) was also observed in RNAi lines [24].

*A. catechu* is one of the most important tropical industrial crops, with important medicinal value including antioxidant, antibacterial and digestion improvement effects [25]. An *A. catechu* survey in Hainan province (unpublished data) demonstrated that the average fruit setting rate is less than 12%, i.e. more than 88% of the total *A. catechu* fruitlets were abscised. However, a few individuals with fruit setting rate more than 50% were observed, indicating the variation of fruitlet abscission percentage in *A.catechu*. Our previous data showed that *A. catechu* fruitlets began to drop on the 10^th^ day after pollination, and experienced a peak of abscission from the 3^rd^ to 4^th^ week after pollination [4]. However, the cross-talk of environmental cues and abscission signals, and the molecular regulatory mechanism concerning fruitlet abscission remains largely unknown. Recently, several transcriptomic and metabolomic data concerning fruit abscission have been obtained from diverse fruit trees, including sweet orange (*Citrus sinensis*) [26], apple (*Malus domestica*) [27] and olive [28]. Here, we performed a combination analysis of phytohormone and transcriptome in *A. catechu* to provide insights into fruitlet abscission. An in-depth understanding of the abscission mechanism will facilitate future fruit production improvement and management methods [9, 29] and help develop molecular markers for genetic breeding of fruit trees.

## Results

### Morphological features of *A. catechu* fruitlets

**Fig. 1:**
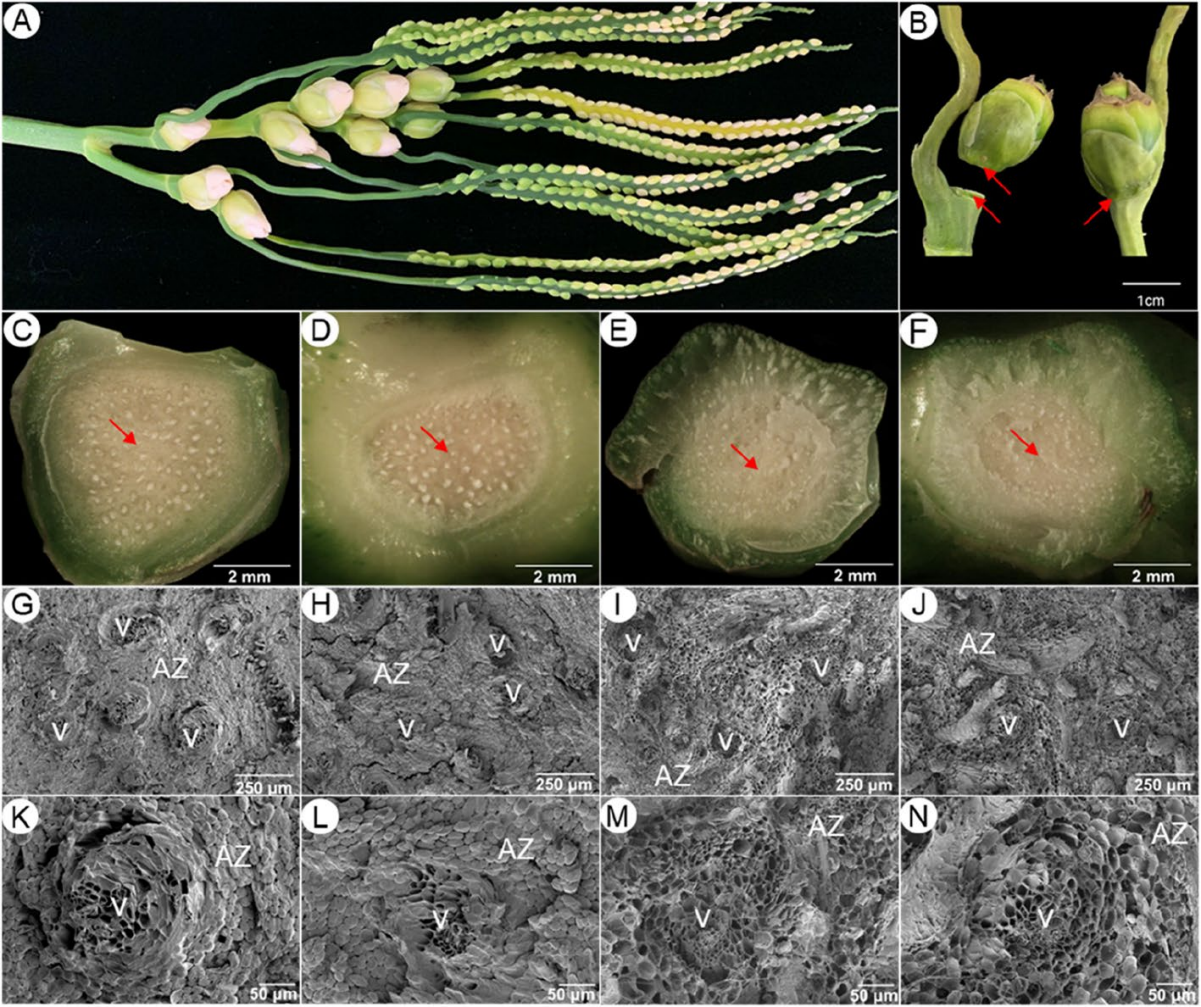
Morphological features of *A. catechu* fruitlet and abscission zone (AZ), **A** Inflorescence of *A. catechu*; **B ***A. catechu* fruitlets 18 days after pollination (left, naturally dropping fruitlet; right, non-abscised fruitlet); **C** Proximal end of AZ in naturally abscised fruitlet; **D** Distal end of AZ in naturally abscised fruitlet; **E** Proximal end of AZ in artificially removed fruitlet; **F** Distal end of AZ in artificially removed fruitlet; **G** and **K** Cell architecture of the proximal end of AZ in naturally abscised fruitlet (at different magnification); **H** and **L** Cell architecture of distal end of AZ in naturally abscised fruitlet (at different magnification); **I** and **M** Cell architecture of proximal end of AZ in artificially removed fruitlet (at different magnification); **J** and **N** Cell architecture of distal end of AZ in artificially removed fruitlet (at different magnification). v: vascular bundles, AZ: abscission zone. The red arrow refers to the AZ cells

*A. catechu* is a monoecious and cross-pollinated species. Surrounding bracts wrap the inflorescences of *A. catechu* at the early developmental stage, and the spikes appear after the bracts split open (Fig. 1A). A large portion of fruitlets will drop in 2–3 weeks after pollination (Fig. 1B). Fruitlets that abscised naturally or about to abscise (dropped by gentle touching) showed a characteristic abscission scar in the abscission zone (AZ). Moreover, the vascular bundles in naturally abscised fruitlets presented closed state (Fig. 1C and D). While the non-abscised fruitlets, forcibly removed, exhibited obvious fracture marks and uneven tissue in AZ, and the vascular bundles were open and broken (Fig. 1E and F). The cell architecture in the fracture surface observed through SEM showed a conspicuous divergence between about-to-abscise and non-abscised AZs. All AZ cells (except those in the vascular bundle) were intact, enlarged and flat round in the naturally abscised fruitlets (Fig. 1G-H, K-L), forming the abscission structure, which is easy to shed spontaneously or by gentle touch. On the contrary, forcible removal of fruitlets will result in broken cells in the AZ fracture plane. Therefore, many broken and hollow cells can be observed in the AZ of the fracture surface (Fig. 1I-J, M–N).

### Variation of phytohormone levels during *A. catechu* fruitlet abscission

**Fig. 2:**
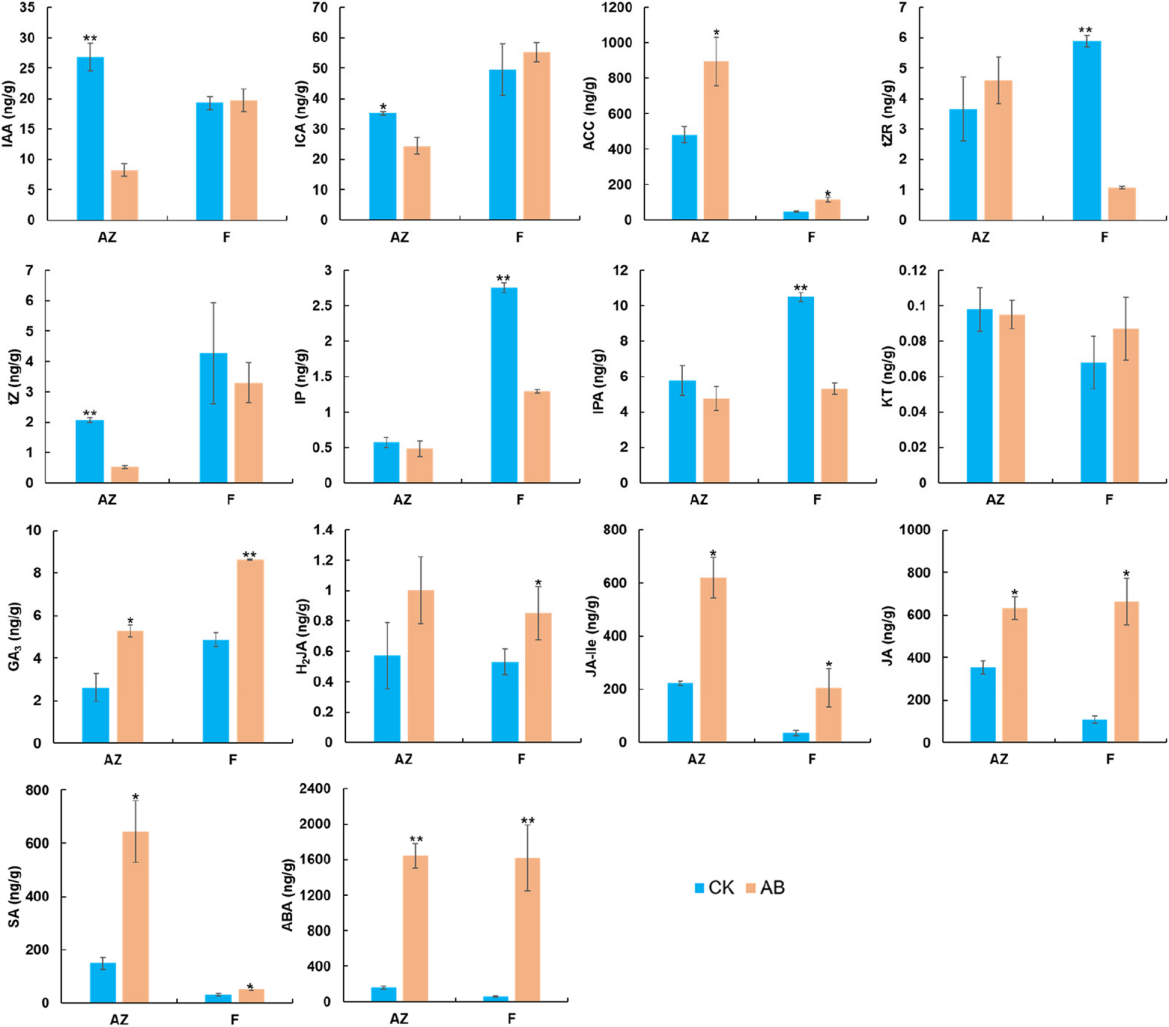
Endogenous phytohormone levels in the AZs (AZ) and fruitlet parts (F) of AB and CK. IAA, indole-3-acetic acid; ICA, indole-3-carboxaldehyde; ACC, 1-Aminocyclopropanecarboxylic acid; tZR, trans-zeatin-riboside; tZ, trans-zeatin; IP, N6-isopentenyladenine, IPA, isopentenyl adenosine; KT, kinetin; GA_3_, gibberellin A3; H2JA, dihydrojasmonic acid; JA-Ile, N-jasmonic acid isoleucine; JA, jasmonic acid; SA, salicylic acid; ABA, abscisic acid; AZ, abscission zone; AB, about-to-abscise fruitlet; CK, non-abscised fruitlet. Error bars represent the SD of three replicates. Significant differences compared to CK for each organ were determined using Student's t-test: *P < 0.05, **P < 0.01

To reveal the role of phytohormone in *A. catechu* fruitlet abscission, liquid chromatography-mass spectrometry (LC–MS) was performed in AZ of both about-to-abscise (AB) and non-abscised (CK) fruitlets to detect endogenous phytohormone levels. Results revealed that in AZ, the levels of auxin, including indole-3-acetic acid (IAA) and indole-3-carboxaldehyde (ICA), were significantly lower in AB than in CK, At the same time, no significant difference was observed between fruitlet parts collected from AB and CK samples. For cytokinin, the trans-zeatin (tZ) level in AZ was significantly lower in AB than in CK, while no difference was observed on trans-zeatin-riboside (tZR), N6-Isopentenyladenine (IP) and isopentenyl adenosine (IPA). On the contrary, the tZ level was unchanged in the fruitlet parts of AB and CK, while the tZR, IP and IPA levels were significantly lower in the fruitlet part of AB than in CK. In AZ, the levels of GA_3_, JA-Ile, JA, SA and ABA were significantly higher in AB than in CK, while a similar varying tendency was also found in the fruitlet part. In addition, the content of 1-amino cyclopropane 1-carboxylic acid (ACC), the intermediate of ethylene biosynthesis, was significantly elevated in AB of both AZ and fruitlet part, while the kinetin (KT) levels were not changed in both AZ and fruitlet part (Fig. 2).

### Identification of DEGs between AZs abscising and non-abscised fruitlets based on transcriptome analysis

The distinction between about-to-abscise and non-abscised fruitlets at morphological, cytological and phytohormone levels indicated that different molecular events exist in these tissues. Therefore, transcriptome analysis was performed in A. catechu AZs of abscising and non-abscised fruitlets. A total of 257 M clean reads were obtained and 37 M to 42 M reads were mapped into the Areca genome [30]. The number of unique matching reads used for subsequent analysis in different samples is from 34 to 39 M, and the matching rate of each sample is more than 87% (Table S2). These results suggest that the sequencing quality is sufficient for subsequent analysis. The transcriptome data has been deposited into the China National Center for Bioinformation with the code CRA007290 (https://ngdc.cncb.ac.cn/search/?dbId=&q=CRA007290).

Totally 19,845 and 20,474 unigenes were identified in AB and CK, respectively. Among them 610 and 1239 genes were specifically expressed in AB and CK, respectively (Fig. S1, Additional file 3). According to the criterion FDR < 0.05 and |log_2_FC|> 1, a total of 1239 DEGs were identified, including 522 up-regulated genes and 717 down-regulated ones (AB vs. CK) (Fig. S2).

**Fig. 3:**
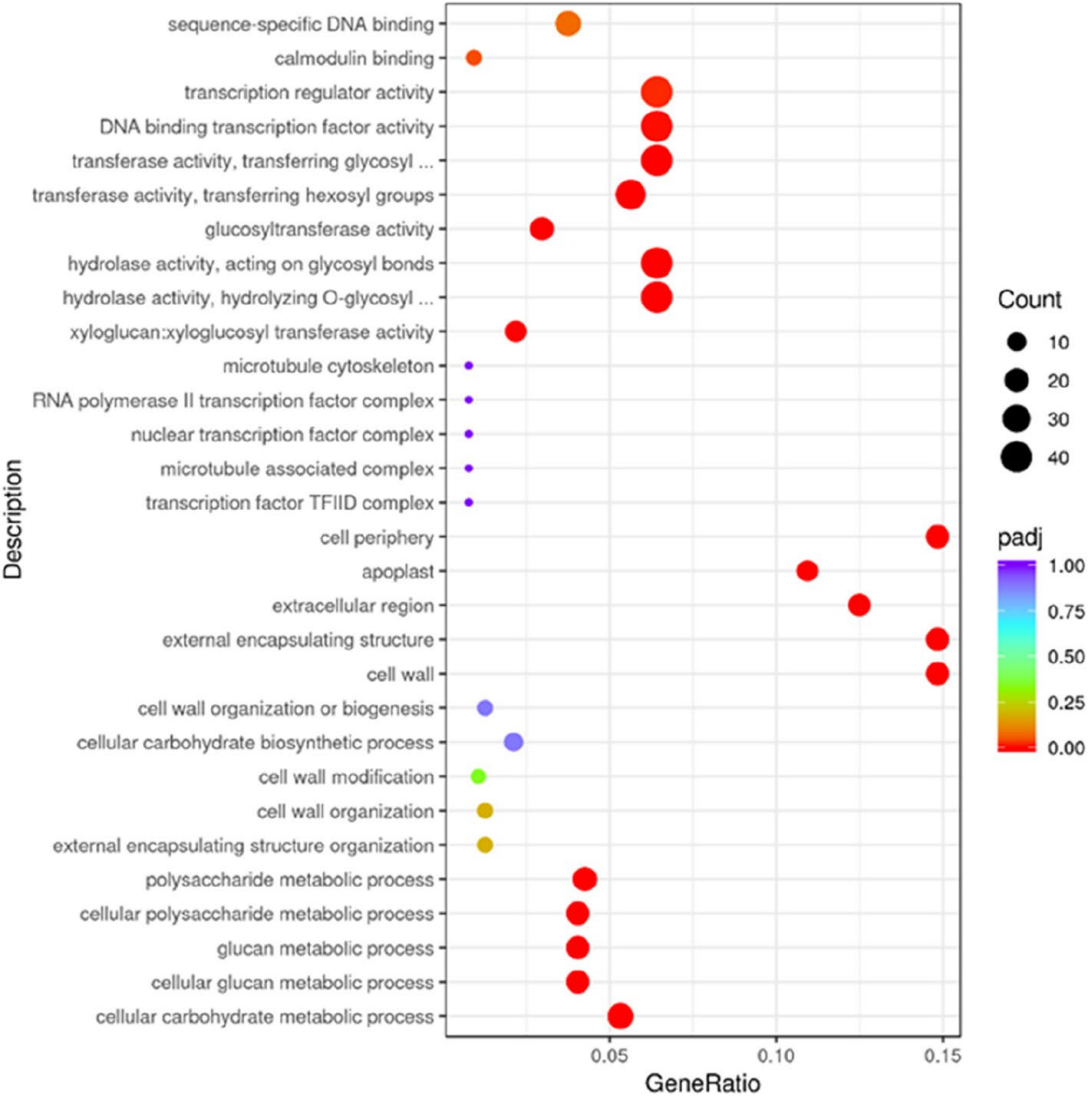
Top 30 different GO terms enriched in AB and CK. The *p-*value cutoff of 0.05 calculated by the FPKM of all genes in corresponding GO terms was used to select enriched GO terms

The identified DEGs were analyzed against the gene ontology (GO) database to determine the biological functions. The top 30 GO terms containing most DEGs are shown in Fig. 3. Most DEGs enriched in the molecular function category involved in DNA-binding transcription factor activity, transcription regulator activity, transfer activity, and transferring glucose groups. Most genes were down-regulated (57.9%) in AB samples, indicating that the expression levels of many genes were inhibited during fruit abscission. In the cell components category, a large number of DEGs were enriched in cell wall, external enveloping structure, and cell circumference pathways, suggesting that cell wall modification is a key event during fruit abscission (Fig. S3). KEGG (Kyoto Encyclopedia of Genes and Genomes) analysis demonstrated that most DEGs were enriched in six metabolic pathways, including starch and sublime metabolism, phenylpropanoid biosynthesis, MAPK signaling pathway, plant hormone signal transformation, galactose metabolism and cysteine and methionine metabolism (Fig. S4).

### The expression patterns of genes related to phytohormone biosynthesis and signal transduction

There are 33 genes related to auxin and ethylene were expressed in the AZ of AB fruitlets. Among them, 7 genes encoding auxin efflux carrier-like protein (PIN-likes 7), auxin response factor (ARF) and Small Auxin Upregulated RNA (SAUR) protein were significantly down-regulated in AB, while 10 genes encoding AP2/ERF transcription factor, ethylene receptor (ETR), ethylene response sensor (ERS) and ethylene insensitive (EIN) were uniformly up-regulated in AB.

**Fig. 4:**
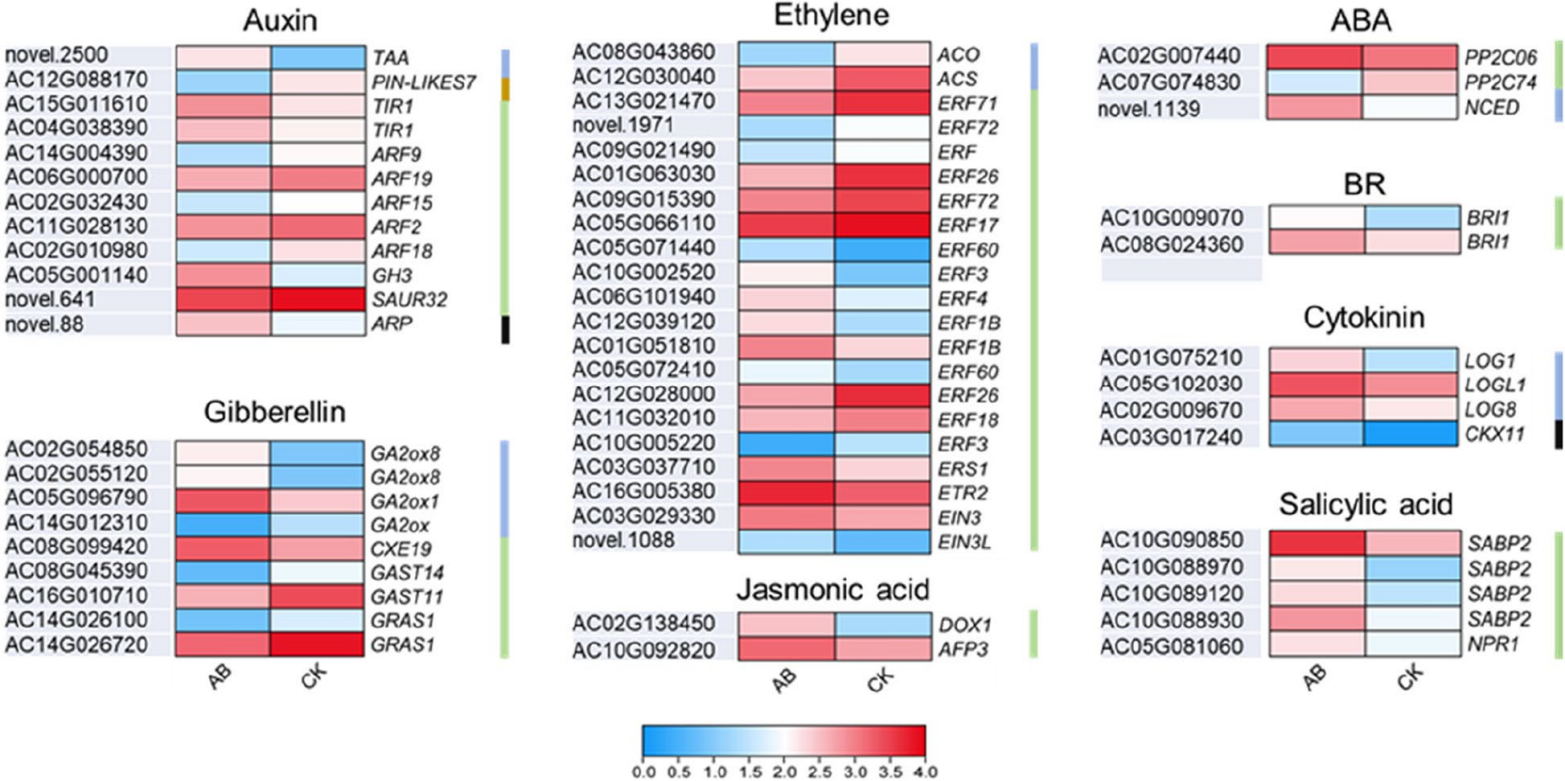
Expression profilings of genes related to phytohormone biosynthesis, signaling, and metabolism in AB and CK. Blue, green, yellow and black vertical indicate genes involved in phytohormone biosynthesis, signaling, transport and degradation, respectively. Gene expression levels are indicated with color bars

As for other phytohormones, 4 genes encoding gibberellin-regulated protein (GAST) and gibberellin-responsive protein (GRAS) were consistently down-regulated, 3 genes encoding cytokinin riboside 5'- monophosphate phosphohydrolase (LOG) related to cytokinin synthesis were significantly up-regulated, while a gene encoding cytokinin oxidase/dehydrogenase (CKX11) was down-regulated, which is the enzyme that catalyses the catabolism of cytokinins to inactive product [31]. Five genes encoding salicylic acid binding protein (SABP), an important esterase determining salicylic acid level. The regulatory protein (NPR1), two genes encoding alpha-dioxygenase (DOX) and Ninja-family protein (AFP 3) that related to disease resistance were all largely up-regulated in AB. In addition, 2 of 3 genes encoding protein phosphatase 2C (PP2C) and 9-cis-epoxycarotenoid dioxygenase (NCED) that related to ABA synthesis, and 2 genes encoding brassinosteroid receptor protein (BRI1) showed significantly higher expression in AB (Fig. 4). The expression patterns of phytohormone related genes were approximately consistent with the phytohormone levels in AB and CK, reinforcing that *A. catechu* fruitlet abscission is under strict regulation of phytohormones and related genes.

### Expression patterns of genes involved in cell wall biosynthesis, degradation, loosening and modification

**Fig. 5:**
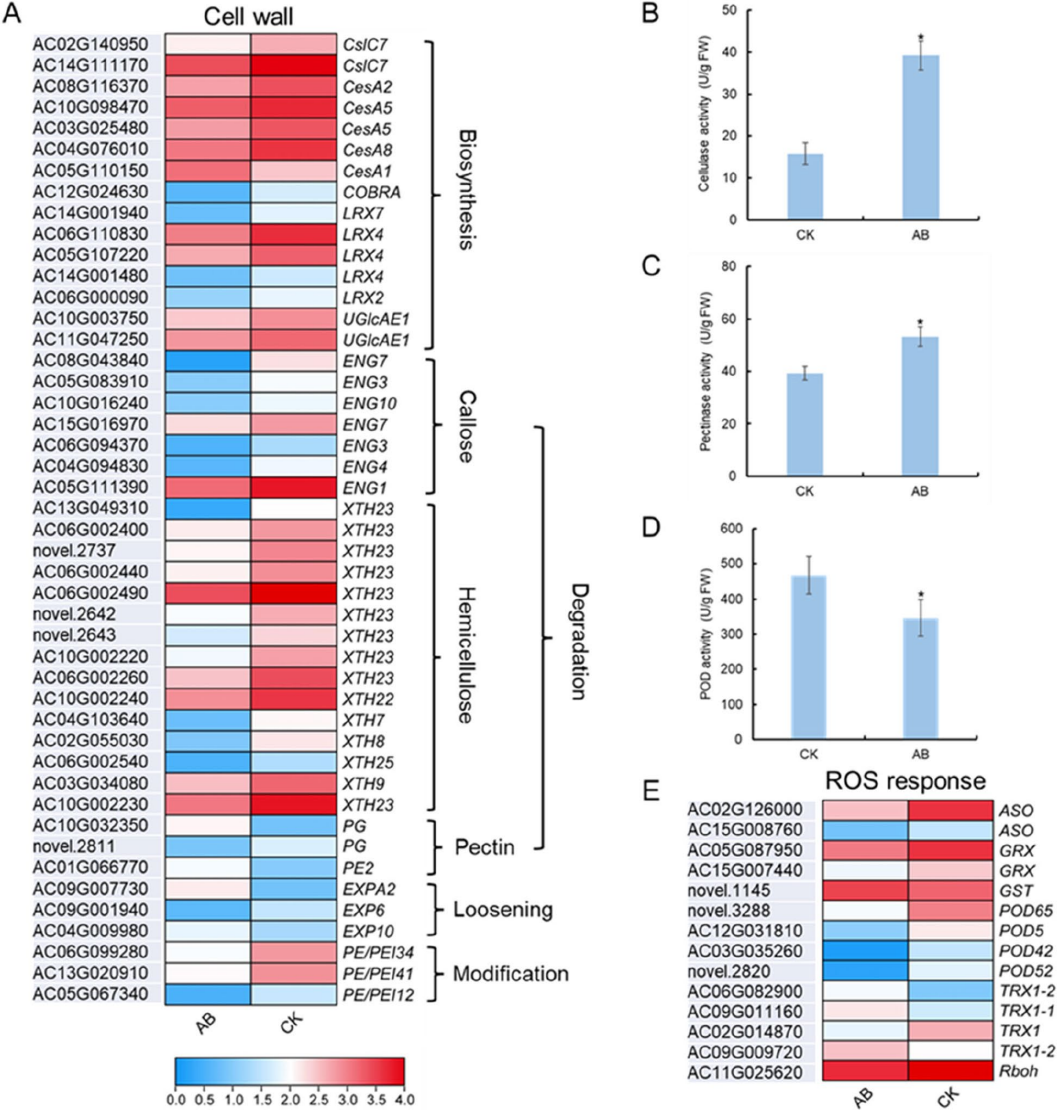
Expression profiling of genes and enzyme activity related to cell wall modification and ROS response in the AB and CK. **A** Expression profiling of genes related to cell wall modification; **B**-**D** Comparison of cellulase (**B**), pectinase (**C**) and POD activities between AB and CK; **E** Expression profiling of genes related to ROS response

A total of 46 genes related to cell wall formation or degradation have been identified, including genes involved in cell wall biosynthesis (15), degradation (25), loosening (3) and modification (3). Among them, all the 15 genes involved in cell wall biosynthesis, except for *AcCesA1* showed uniform down-regulation in AB, including genes encoding cellulose synthase, leucine-rich repeat extensin-like protein, UDP glucuronate 4-isomerase, UDP glucuronate 4-epimerase and xyloglucan glucosyltransferase. On the contrary, genes involved in cell wall degradation, including 1gene encoding polygalacturonases (PGs) and 2 genes encoding expansins (EXPs) were significantly up-regulated in AB. In addition, three *PE/PEIs* genes involved in cell wall modification were significantly down-regulated in AB (Fig. 5A). These results indicated that cell wall biosynthesis was obstructed and the connection between cells was loosen, which is a cue for cell wall to degrade. Furthermore, the enzyme activity of cellulose and pectinase was verified to be significantly higher in AB than that in CK (Fig. 5B and C).

### Gene related to reactive oxygen species (ROS) scavenging and lignin biosynthesis might also affect abscission regulation

Generally, the ROS level will increase in separating organs, and the enzymes with scavenging ROS function will be activated in corresponding regions. A total of 14 genes related to ROS scavenging were identified as DEGs between AB and CK. However, 4 of them encoding peroxidase (POD) were suppressed in AB, while 1 of them encoding glutathione S-transferase (GST) showed prominently up-regulation in AB (Fig. 5E). Correspondingly, the POD activity decreased in AB (Fig. 5D). In addition, the expression of a gene encoding L-ascorbate oxidase (AO), a key enzyme in the pathway of vitamin C synthesis, was also suppressed in AB.

### Candidate transcription factors involved in fruitlet abscission

**Fig. 6:**
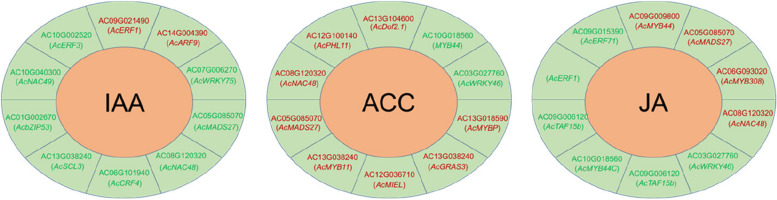
Top 10 correlation network of transcription factors and phytohormone contents. The red and green fonts represent positive and negative correlations, respectively

Transcription factors (TF) play an important role in plant organ abscission. A total of 11 types of transcription factors were identified as potential regulators participating in fruitlet abscission, including MYB, NAC, ERF, WRKY, ZF, ARF, bHLH, bZIP, GRAS, KNOX and MADS. MYB and NAC showed the most different expression pattern between AB and CK, followed by ERF, WRKY and ZF. 12 MYB genes and 10 NAC genes showed up-regulation in AB [4]. For the ZF family, five were up-regulated and three genes were down-regulated in AB. These genes encoding TFs may be critical regulators and important molecular markers for the process of fruitlet abscission in *A. catechu*. Then, the correlation between identified DEGs encoding TFs and eight genes phytohormone was determined. The IAA, ACC, JA, SA and ABA levels were positively or negatively related to *AcERF1* (AC09G021490), *AcWRKY46* (AC03G027760), *AcNAC48* (AC08G120320) and *AcMADS27* (AC05G085070) (Figs. 6 and S5). It was noticed that the *AcERF1* (AC09G021490) is positively related to IAA contents but negatively related to JA contents, and *AcNAC48* (AC08G120320) is positively related to ACC, JA, SA and ABA contents but negatively related to IAA and tZ contents (Figs. 6 and S5), indicating the antagonistic relationship between these phytohormones during fruitlet abscission.

### Validation of the expression patterns of selected DEGs by qPCR

**Fig. 7:**
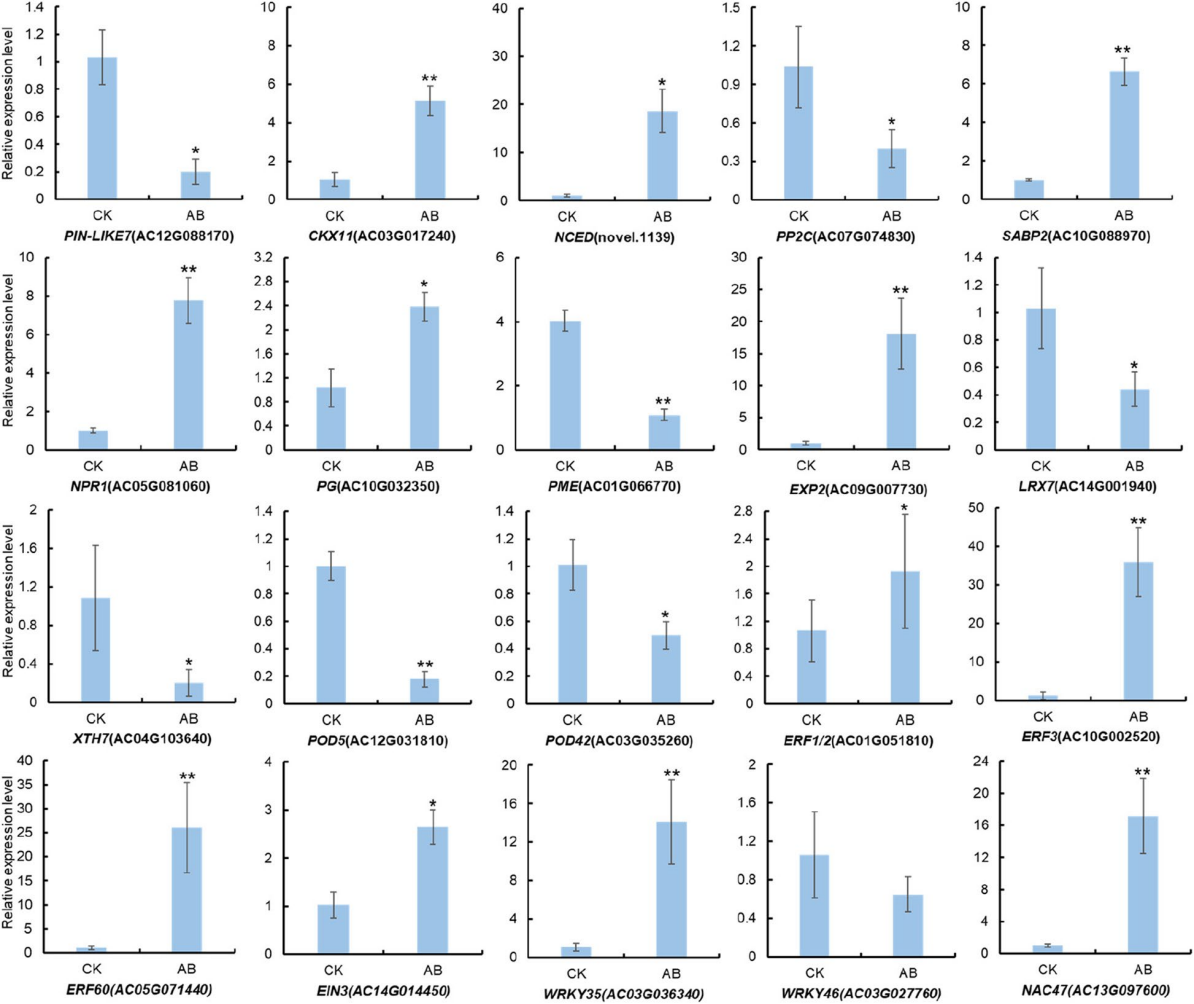
RT-qPCR analysis of the expression of 20 DEGs between AB and CK. * and ** indicate significant differences in comparison with values at CK at *P* < 0.05 and *P* < 0.01, respectively (*t*-test), and the error bars indicate the standard deviations

To verify the expression patterns of candidate genes, 20 DEGs with potential functions were selected, including six genes related to phytohormone synthesis, transport and signal transduction, five genes related to cell wall modification, two genes related to ROS, and seven genes encoding transcription factors. The expression levels of these genes in AB and CK were detected using RT-qPCR (Fig. 7). High consistency was verified between RNA-Seq and RT-qPCR results for the candidate unigenes (*R*^2^ = 0.8184), indicating that the expression data obtained by RNA-Seq was reliable (Fig. S6).

## Discussion

All AZ cells (except for those in the vascular bundle) were intact in the naturally abscised fruitlets. This observation reinforced that the cells distributed at AZ's proximal and distal ends were completely separated [32]. On the contrary, the AZ cells in the fracture surface formed by external force were broken, indicating that the cell wall degradation process required to separate of adjacent cells at the proximal and distal ends has not been completed in these tissues. In addition, vascular bundles with intact structures were observed in both naturally abscised and artificially removed fruitlets. The marginal part of the vascular bundles in naturally abscised fruitlet was partially closed, indicating that material transportation was cut down or ceased in this region. Differently, densely distributed granular materials were observed in the vascular bundles of artificial removed fruitlets, implying continuous material input in these tissues.

There are many abiotic and biotic signals have been reported to regulate organ abscission, including temperature, light, water and nutrient supply, and pathogen, etc. [5, 9, 33–35]. However, extensive documents manifest that these signals exert the abscission function mediated by phytohormones. In the AZ of *A. catechu* fruitlets, we noticed that the auxin level was significantly decreased in AB, while this difference was not observed in other parts of the fruitlets (Fig. 2), indicating that the polar translocation of auxin was specifically inhibited in AB. Auxin plays a key role in organ abscission regulation. It is generally thought that the initiation of abscission is in dormant state, and will not be triggered if a continuous auxin flow is present in AZ [4]. The direction of auxin polar transport is from fruit to AZ. A common decreasing pattern of auxin distribution from other parts to AZ was observed in the tomato pedicel and rose petal abscission [36, 37]. In tomato, silencing of a *PIN1* gene (PIN-FORMED1) retained auxin in ovary and reduced the auxin level in AZ, thus aggravating pedicel abscission [38]. Therefore, polar auxin transport regulates organ abscission by altering the auxin level balance. Interfering polar auxin transport led to a reverse distribution of auxin level, resulting in floral organ abscission in yellow lupine (*Lupinus luteus* L.) [39]. It was noticed that a gene encoding an auxin polar transport vector, *PIN-likes7*, was specifically down-regulated in AB fruitlets, indicating that similar regulation also occurs in *A. catechu*.

On the other hand, depletion of auxin will enhance the sensitivity of AZ to ethylene, thus initiating the abscission process [40–42]. Ethylene has been verified to affect auxin levels by inhibiting auxin transport [43]. The role of ethylene in abscission has been extensively documented, and genes involved in ethylene biosynthesis and signal transduction were identified as key regulators of organ abscission in diverse plant species. The Arabidopsis mutants, such as *etr1* (ethylene resistant 1), *ein2* (ethylene insensitive 2), *ein3* (ethylene insensitive 3) and *ers2* (ethylene response sensor 2) all delayed floral organ shedding to different degrees [8, 44, 45]. Overexpression of an *LcEIL2/3* gene encoding a transcription factor involved in ethylene signal transduction pathway accelerated floral organ abscission in both wild-type *Arabidopsis* and *ein3 eil1* mutants. The LcEIL2/3 transcription factor mediated ethylene-induced fruitlet shedding by regulating the expression of *LcACS1/4/7*, *LcACO2/3*, *LcCEL2/8* and *LcPG1/2*, thus changing ethylene biosynthesis and triggering cell wall degradation [46]. The gene-encoding rate-limiting enzymes ACO (ACC oxidase) and ACS (ACC synthase) of ethylene biosynthesis were significantly suppressed in AB. By contrast, the genes involved in ethylene signal transduction, including *ERS*, *ETR* and *EIN*, were significantly up-regulated in AB (Fig. 4). This result manifests that the changes in ethylene biosynthesis was occurred during fruitlet abscission in *A. catechu*. The synthesis and release of ethylene is critical for abscission signal transduction. However, continuous release of ethylene may have side effects other than fruitlet abscission induction. Therefore, the inhibition of ethylene synthesis indicated by the down-regulation of related genes might be programmed by the maternal plant to achieve precise control of fruit abscission.

Cytokine (CTK) was reported as an abscission promotor, but a high concentration of CTK inhibits abscission [47]. In cotton (*Gossypium hirsutum*), exogenous treatment of thidiazuron or ethephon promoted the degradation of endogenous cytokinin, while enhanced the production of endogenous ethylene, and resulted in cell wall destruction and cell separation [14]. Therefore, CTKs may participate in abscission as a mediator. A gene encoding cytokinin oxidase/dehydrogenase (CKX) and three genes encoding LONELY GUY (LOG) were significantly up-regulated in AB (Fig. 4). CKX catalyzes the catabolism of cytokinin to inactive products [31], thus the activation of CKX results in the reduction of endogenous cytokinin in plant cells [48, 49]. The *LOG* gene encodes cytokinin nucleoside 5'-monophosphate phosphoribosyl hydrolase, directly converting the non-active cytokinin nucleoside into bioactive product isopentene adenine (IP). The trans-zeatin (tZ) level in AB was significantly lower than that in CK (Fig. 2). A possible explanation is that the up-regulation of *LOG* genes in AB enhanced the formation of IP. Meanwhile, the up-regulation of *CKX* gene induced the rapid decomposition of CTK.

The ABA level is prominently elevated in many plants before floral organ or fruit abscission [50, 51]. Therefore, ABA is widely adopted as a key factor directly determining cell separation. However, increasing studies indicated that ABA acts on organ senescence rather than promotes abscission directly. The effect of ABA on abscission seems to depend on the interaction with other phytohormones, but not by itself [9]. For instances, exogenous ABA treatment did not change organ abscission in citrus, blue flax (*Linum lewisii*) and montbretia (*Crocosmia* × *Crocomiiflora*). While endogenous ABA can improve the accumulation of 1-aminocyclopane-1-carboxylic acid (ACC), an ethylene synthesis precursor, promoting ethylene biosynthesis and fruit abscission [52]. The about-to-abscise fruitlets with elevated ABA levels indicated these organs' premature senescence state. In addition, the increased level of salicylic acid (SA), and the expression of a related gene *NPR1* might also accelerate the senescence of fruitlets before abscission in *A. catechu*.

GA participates in organ abscission in diverse plants in an elusive way, and even in opposite manner in different species. For instances, exogenous application of GA can prevent fruit abscission in peach (*Prunus persica*) [10]. GA levels in the ovary was promoted by pollination, thereby inhibited fruit abscission in *Citrus reticulata* [53, 54]. On the contrary, GA_3_ was proven to promote coleoptile and petiole shedding in some legume species [55, 56]. Flower abscission promoted by GA_3_ was also observed in grape (*Vitis vinifera*) [57]. Similarly, exogenous GA_3_ treatment improved the abortion rate of flowers, and the increasing level of GA_3_ in AZ was observed during the shedding process of flowers in *Lupinus luteus* [58]. These divergent results obtained in various plants indicate that the GA-mediated abscission might differ in species and organs. In this study, we noticed that the GA_3_ level is significantly higher in AB than that in CK in both AZ and fruitlet part. However, it cannot be excluded that transport of GA_3_ and consequently high content of this phytohormone in AZ cells can be a stress reaction, which can also induce abscission. Further investigation is needed to clarify the complicated role played by GA in organ abscission.

Our results suggest that JA also play a role in fruitlet abscission in *A. Catechu*. Genes involved in JA signal transduction (*DOX* and *AFP*) were significantly up-regulated, indicating the dynamic changes of JA level in AB. It was found that exogenous MeJA treatment can induce soybean leaf abscission [12], and the cases of fruit abscission in citrus, apple and tomato [13, 14, 59]. A recent study proposed that *SlHB15A* was highly expressed in the flower pedicel abscission zone and induced by auxin in tomato. SlHB15A regulates abscission by depressing JA-isoleucine (JA-Ile) levels by inhibiting the expression of *JASMONATE-RESISTANT1* (*SlJAR1*), a gene involved in JA-Ile biosynthesis [60].

On the other hand, ABA, SA and JA are stress-related phytohormones mainly involved in the typical stress response pathway. SA is involved in the defense response of plants against pathogens. It can trigger the defense response in distal plant parts to protect uninfected tissues [61]. Moreover, SA can regulate the conformation of NPR1 into its active monomeric forms, thereby promote the expression of the *PATHOGENESIS RELATED* (*PR*) gene and subsequent defense responses [62]. Besides SA, the level of JA usually increases in response to pathogen infection, indicating its critical role in plant defense responses. ERF1, ERF2, ERF5 and ERF6 control the expression levels of JA-responsive marker gene *PLANT DEFENSIN 1.2* and provide resistance against necrotrophic pathogens [63]. In addition, the JA-mediated pathway also regulates plant defense against a number of herbivores, such as caterpillars, spider mites, beetles, thrips and mirid bugs [64]. Their accumulation in AB might alter the expression of defense-related genes before the protective layer formation [61]. The expression of these defense-related genes will enhance the strength of cell wall structure in the protective layer. This process is necessary for both maternal tissues and abscised organs [65].

Up to now, there are nine transcription factor families have been reported to be involved in organ abscission regulation, including Aux/IAA, ARF, EIN3, ERF [46, 66–68], MADS-box [69], KNOTTED-LIKE HOMEOBOX, HD-ZIP, DOF and ZF [17, 19, 70, 71]. In *A. catechu*, we identified six genes encoding DOF transcription factor that were significantly up-regulated in AB. Among them, three *AcDof* genes were highly correlated with the fruitlet abscission process [4], especially *AcDof2.1*, which displayed a significant positive correlation with ACC contents (Fig. 6), indicating that the DOF family is critical for *A. catechu* fruitlet abscission. Similarly, *AtZFP2* (zinc-finger protein 2) was expressed explicitly in the sepal AB and induced abscission in *Arabidopsis* [71]. Another gene *AtDof4.7*, belonging to the zinc finger superfamily, can directly inhibit the expression of a polygalacturonase gene associated with cell wall degradation, resulting in floral organs failure to shed normally [17, 18].

For other TF family, ERF1 inhibits BGLA by activating the transcription inhibitor ERF4, thereby inhibiting pectin degradation and petal shedding in Chinese rose [66]. In tomato, SlERF52 regulates the transcription of *SlTIP1;1*, thus increasing the content and permeability of hydrogen peroxide in the cytoplasm to accelerate the process of floral abscission [72]. The litchi gene *LcERF2*, an ethylene-responsive AP2/ERF family member, regulates fruit abscission by directly targeting UDP-glucose-4-epimerase. Overexpression of *LcERF2* promoted fruit abscission and decreased galactose and pectin content in the cell walls of pedicels [68]. Several *ERF* genes were identified as DEGs during fruitlet abscission in *A. catechu* (Fig. 4), and *AcERFs* displayed a better correlation with IAA and JA levels (*r* > 0.9) (Fig. 6). These genes may participate in phytohormone balance and cell wall hydrolysis.

Taken together, fruitlet abscission in *A. catechu* is under the crosstalk of multiple phytohormones. The interaction between auxin and ethylene is critical for determining the fruitlet fate, i.e. abscission or further development. While ABA, JA and SA induced organ senescence, cell wall degradation and protective layer formation [61]. The genes encoding protein products involved in phytohormone biosynthesis and transport, and their upstream transcription factors are key regulators of fruitlet abscission. Several genes encoding TFs, such as *AcERF1*, *AcWRKY46*, *AcNAC48* and *AcMADS27*, might play roles in fruitlet abscission by regulating phytohormone biosynthesis or transport. Due to the fact that all the collected samples were from the later stage of abscission, it's hard to say whether these results are the cause or the outcome of the fruitlets abscission. However, these candidate genes can be adopted as molecular markers for *A. catechu* breeding, and further investigation focusing on their functions will provide new insights into the mechanism of abscission.

## Conclusions

**Fig. 8:**
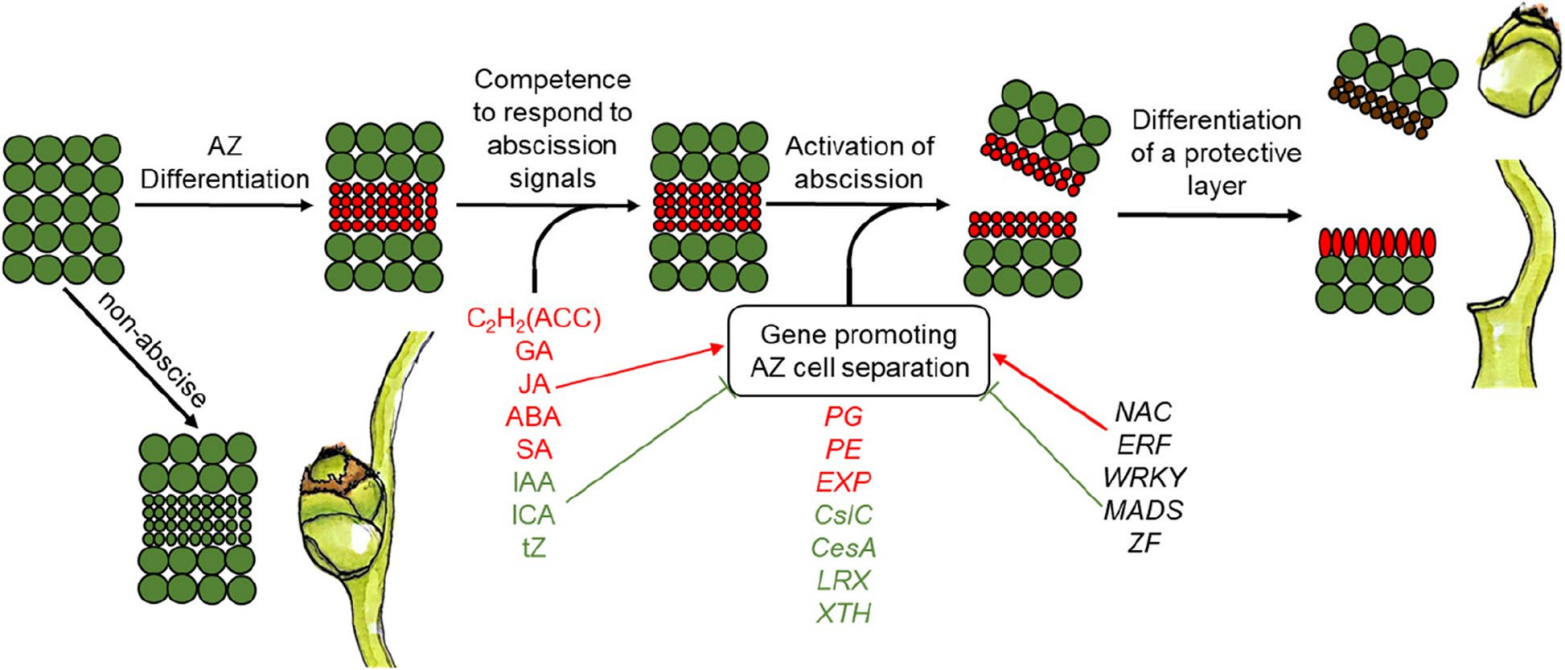
A hypothetical model of fruitlet abscission in *A. catechu*. The possible molecular events to control the *A. catechu* fruitlet abscission based on phytohormone level and transcriptome data. The cells located in the AZ of non-abscise fruitlets will develop into a compact structure (represented by small green circles), while the cells of AZ in about-to-abscise fruitlets will differentiate to a “pre-abscission” type (represented by small red circles). After abscission, the AZ cells in separated fruit will perish (represented by small brown circles), while the AZ cells in maternal plant will form a protective layer (represented by elongated red ellipses). The variation of gene expression levels and phytohormone levels in AZ were indicated with different colors (red for up-regulated and green for down-regulated)

Fruitlet abscission is a bottleneck of the A. catechu industrial. This study detected the variation of phytohormone levels in the AZ of A. catechu fruitlets, and the comparison between "non-abscised" and "about-to-abscise" fruits were analyzed. We proposed that auxin, ethylene and their interaction are critical determinants for abscission. Meanwhile, ABA and CTK act as mediators during the abscission process, while JA and SA may play a critical role in regulating the expression of defense-related genes before the formation of protective layer in the AZ. Several genes encoding enzymes involved in phytohormone biosynthesis and transduction have been identified as key genes of abscission, such as auxin response factor, ethylene receptor, ethylene response sensor, cytokinin oxidase, 9-cis-epoxycarotenoid dioxygenase, etc. The transcription factor families, including NAC, ERF, WRKY, ZF and MADS were identified as candidates involved in abscission. Our results indicated that fruitlet abscission in *A. catechu* evolves a series of processes including AB cell differentiation, cell wall degradation, organ separation and protective layer formation, which are under integrative control of phytohormone and genetic regulation (Fig. 8). This study not only provides a theoretical basis for fruitlet abscission in A. catechu, but also identified many candidate genes or potential molecular markers for further breeding of fruit trees.

## Methods

### Plant materials

Three 8-year-old *A. catechu* trees of the variety Reyan No. 1 grown in the arecanut nursery of the Coconut Research Institute of Chinese Academy of Tropical Agricultural Sciences were used in this study. The permission was obtained from the Agricultural Research, Education and Extension Organization of China to collect plant samples. The specimens were stored at the Coconut Research Institute of Chinese Academy of Tropical Agricultural Sciences. The plants were identified by the first author, Dr. Jia Li.

The samples of fruitlet AZ were collected on the 25th day after the pistillate flowers bloomed. The "about-to-abscise" (AB) and "non-abscised" (CK) parts are defined as the AZ parts that will and will not shed by gentle touching, respectively. The AB samples were collected around 2 mm at each side of the abscission fracture surface, while the CK samples were collected on the tissues 2 mm above and below the calyx side. The collected samples were fixed for cytological observation, and the other group was quickly frozen in liquid nitrogen and stored at -80 °C for RNA extraction and biochemical and physiological measurement, respectively.

### Phytohormone content measurement and enzyme assay

Each sample (three biological replicates) was prepared from 500 mg (Fresh Weight) plant tissue to measure hormone levels. The experiment was carried out according to the method described previously [73]. The hormone contents were measured using a microTOFqorthogonal-accelerated TOF mass spectrometer (Bruker Daltonics, Germany). Cellulase activities were measured using a tissue blotting and gel-diffusion method [74, 75]. The extraction of pectinase was used in the Pectinase test kit (Solarbio, Beijing, China). The POD enzyme activities were determined using the method described by Li et al. [76].

### Transcriptome analysis

Trizol reagent was used to extract extraction of total RNA from three biological replicates of AB and CK. The methods of cDNA library construction, Illumina sequencing and data processing were the same as the description in our previous study [4]. Gene function was annotated based on the following databases: NCBI non-redundant protein sequences (NR), clusters of orthologous (KOG/COG), gene ontology (GO), manually annotated and reviewed protein sequence database (Swiss-Prot), and Kyoto Encyclopedia of Genes and Genomes (KEGG). Gene expression levels were represented using the FPKM method (expected number of fragments per kilobase of transcript sequence per millions base pairs sequenced). The differentially expressed genes (DEGs) were recruited based on False Discovery Rate (FDR) < 0.05 and | log_2_Fold Change|≥ 1. All DEGs were analyzed by GO enrichment using GOseq (1.10.0) [77] and KEGG enrichment using KOBAS software [78].

### Scanning electron microscopy

For SEM analysis, fruitlet AZ from “about-to-abscise” and “non-abscised” parts of the 25th day after pistillate flowers bloomed were collected, fixed in 4% (w/v) glutaraldehyde in 0.05 M potassium phosphate buffer (pH 7.4), and then rinsed four times in the buffer. After dehydration in a graded ethanol series, the abscission samples were critical-point dried in liquid CO_2_. Samples were sputter coated with gold, and were viewed at 5 kV on a Hitachi SU8100 Field Emission Scanning Electron Microscope. For the morphology analysis, the samples were collected from different individuals at least three times.

### qPCR analysis

The extracted RNA of AB and CK samples were reversely transcripted into cDNA using the PrimeScript™ RT reagent Kit for qPCR (TaKaRa, Japan). The qPCR reaction was performed using the PowerUp™ SYBR™ Green Master Mix (Applied Biosystems, United States) in an Applied Biosystem 7500 real-time PCR system. An *A. catechu* gene, *AcActin* (CL9155.Contig7) [79] was used as the internal control for data normalization. Primers used in qPCR are shown in Supplementary Table S 1. The relative expression fold of each sample was calculated by its C_T_ value normalized by the reference gene using the 2^*−ΔΔCT*^ method. All the results were derived from three independent biological replicates with internal repeats.

### Correlation analysis of phytohormone and transcription factors

The DEGs from transcription factors involved in the abscission of fruitlets were subjected to association analysis with three types of differentially accumulated phytohormone. Correlation analysis was performed by calculating the Pearson correlation coefficient (PCC) between the phytohormone contents and transcriptional changes, and the screening criterion was PCC ≥ 0.80 or ≤ -0.80.

### Statistics

All experiments were carried out in at least three biological replicates. Student's t-test was used to assess the statistical significance of the results, as described in corresponding sections of methods and figure legends.

### Supplementary Information

**Additional file 1:** **Table S1.** Sequence information of the primers used in this study. **Table S2.** Statistics of digital transcript abundance library sequencing.

**Additional file 2:** **Figure S1.** Venn diagram of differentially expressed genes (DEGs) in AB and CK. **Figure S2.** The counts of DEGs in AB and CK. **Figure S3.** GO classification of DEGs between AB and CK. The X-axis represents the functional classification, and the Y-axis represents the number of genes enriched into related GO terms. BP, biological process; CC, cell component; MF, molecular function. **Figure S4.** Top 20 enriched KEGG pathways evolving DEGs between AB and CK. The X-axis represents the gene ratio (gene ratio = number of enriched genes / number of all genes in a certain pathway), and the Y-axis represents different KEGG pathways; the size of the bubble is proportional to the number of genes enriched in the KEGG pathway; different colors represent the Q-value of enrichment. **Figure S5.** Top 10 correlation network of transcription factors and phytohormone contents. The red and green fonts represent positive and negative correlations, respectively. **Figure S6.** Correlation of gene expression results. The x-axis represents the value of Log_2_ FPKM and the y-axis represents the value of Log_2_ normalized expression level. Blue round dot represent AB. Orange round dot represent CK. R^2^ value represent the correlation between RNA-seq and qPCR results.

**Additional file 3.** DEGs between CK and AB.

## Data Availability

The transcriptome data has been deposited into the China National Center for Bioinformation with the code CRA007290 (https://ngdc.cncb.ac.cn/search/?dbId=&q=CRA007290).
