# Supplementary material for: Phytohormones and candidate genes synergistically regulate fruitlet abscission in Areca catechu L
Source: BMC Plant Biol. 2023 Nov 3;23:537. doi: 10.1186/s12870-023-04562-8 (PMC10623784; doi:10.1186/s12870-023-04562-8)
Supplement: Supplementary file 1 — Additional file 1: Table S1. Sequence information of the primers used in this study. Table S2. Statistics of digital transcript abundance library sequencing. [file 12870_2023_4562_MOESM1_ESM.docx]

Table S1. Sequence information of the primers used in this study.

| Gene ID | Gene name | Primer (5’ to 3’) |
| --- | --- | --- |
| *AC14G014450* | *AcEIN3-F* | gatggttttgaagatgcccatgct |
|  | *AcEIN3-R* | tgctgcttaccttgaacaggttgg |
| *AC03G017240* | *AcCKX11 -F* | tcgagtggggaatccggcgaca |
|  | *AcCKX11 -R* | tcgagctccaacacgttggca |
| *AC01G051810* | *AcERF1/2 -F* | cggcaccaaagagcagagctacc |
|  | *AcERF1/2 -R* | tcgagaacgccgcctggtcgta |
| *AC05G071440* | *AcERF60-F* | ttcggcgatcccggaggaggtt |
|  | *AcERF60 -R* | cgtgaagtccaggcactgcatc |
| *AC01G066770* | *AcPE -F* | cagaactgcaacattttcgccaga |
|  | *AcPE -R* | gaacgaaccgttctgcgcacc |
| *AC05G081060* | *AcNPR1 -F* | aggtggaggaatttttcgcgatcc |
|  | *AcNPR1-R* | cgtgcaaacgtcgggctcca |
| *novel.1139* | *AcNCED-F* | cggccatcacttcttcgacgggga |
|  | *AcNCED-R* | gagctcaccgatagccttgggga |
| *AC13G097600* | *AcNAC47-F* | tacccgaacgggatccggccaa |
|  | *AcNAC47-R* | ttcacgcccttgggaggtct |
| *AC14G001940* | *AcLRX-F* | gaacaatgggctcaactcttgcct |
|  | *AcLRX-R* | gagccacatccagctgctccaa |
| *AC09G007730* | *AcEXP2-F* | gaacaatgggctcaactcttgcct |
|  | *AcEXP2-R* | tgtcgttggggagggcgtagttc |
| *AC10G002520* | *AcERF3-F* | ttggggacctttgagactgccga |
|  | *AcERF3-R* | cagctttgccatgaggcttggtga |
| *AC10G032350* | *AcPG-F* | acgacgtggtaagtttcggagcca |
|  | *AcPG-R* | ccatggaagctggcttggct |
| *AC12G031810* | *AcPOD5-F* | tggggcacataccattggccggt |
|  | *AcPOD5-R* | tggtactcccctgggggcattgt |
| *AC03G035260* | *AcPOD42-F* | agggatggcattgttgcgcttg |
|  | *AcPOD42-R* | ccaatggcactaaacttgtccagg |
| *AC12G088170* | *AcPIN-LIKES7-F* | cctacagtctgatgcggaaatc |
|  | *AcPIN-LIKES7-R* | gaagcaaagcttcttgatccctgt |
| *AC07G074830* | *AcPP2C-F* | ttgcatgcagccaatgttggtgac |
|  | *AcPP2C-R* | gcgacaactcacatatcccccaga |
| *AC10G088970* | *AcSABP2-F* | gtccccaattcatgtcactcaagc |
|  | *AcSABP2-R* | gtagaccacatcaaccgatcca |
| *AC04G103640* | *AcXTH7-F* | cttgttcgggcgtgtgagca |
|  | *AcXTH7-R* | ggaccgtgtaaggatgtccactcc |
| *AC03G036340* | *AcWRKY35-F* | gcccacaagaaccatcccctcaga |
|  | *AcWRKY35-R* | tcttgggagcacttgggtggacat |
| *AC03G027760* | *AcWRKY46-F* | tggagttggatcacttggcact |
|  | *AcWRKY46-R* | tggagttggatcacttggcact |
| *CL9155.Contig7* | *AcActin-F* | attcaggtgcccggaggtcctctt |
|  | *AcActin-R* | gggaacatggttgatcccccacta |

Table S2. Statistics of digital transcript abundance library sequencing.

| Sample | Total Reads | | Total mapped reads | Unique mapped reads |
| --- | --- | --- | --- | --- |
| CK1 | 45,184,438 | 42,748,564 (94.61%) | | 39,732,402 (87.93%) |
| CK2 | 42,681,970 | 40,008,612 (93.74%) | | 37,400,896 (87.63%) |
| CK3 | 42,464,122 | 40,042,900 (94.30%) | | 37,455,544 (88.21%) |
| AB1 | 45,536,868 | 42,874,256 (94.15%) | | 39,769,664 (87.34%) |
| AB2 | 42,595,730 | 40,567,439 (95.24%) | | 37,481,983 (87.99%) |
| AB3 | 39,194,982 | 37,303,704 (95.17%) | | 34,175,749 (87.19%) |

*Number and percentage of reads mapped onto *A. catechu* genome (NCBI accession number: CNP0000517). CK and AB mean the “non-abscised” and “about-to-abscise” parts of AZ.
