# Supplementary material for: Phytohormones and candidate genes synergistically regulate fruitlet abscission in Areca catechu L
Source: BMC Plant Biol. 2023 Nov 3;23:537. doi: 10.1186/s12870-023-04562-8 (PMC10623784; doi:10.1186/s12870-023-04562-8)
Supplement: Supplementary file 2 — Additional file 2: Figure S1. Venn diagram of differentially expressed genes (DEGs) in AB and CK. Figure S2. The counts of DEGs in AB and CK. Figure S3. GO classification of DEGs between AB and CK. The X-axis represents the functional classification, and the Y-axis represents the number of genes enriched into related GO terms. BP, biological process; CC, cell component; MF, molecular function. Figure S4. Top 20 enriched KEGG pathways evolving DEGs between AB and CK. The X-axis represents the gene ratio (gene ratio = number of enriched genes / number of all genes in a certain pathway), and the Y-axis represents different KEGG pathways; the size of the bubble is proportional to the number of genes enriched in the KEGG pathway; different colors represent the Q-value of enrichment. Figure S5. Top 10 correlation network of transcription factors and phytohormone contents. The red and green fonts represent positive and negative correlations, respectively. Figure S6. Correlation of gene expression results. The x-axis represents the value of Log2 FPKM and the y-axis represents the value of Log2 normalized expression level. Blue round dot represent AB. Orange round dot represent CK. R2 value represent the correlation between RNA-seq and qPCR results. [file 12870_2023_4562_MOESM2_ESM.docx]

**Supplementary Material**


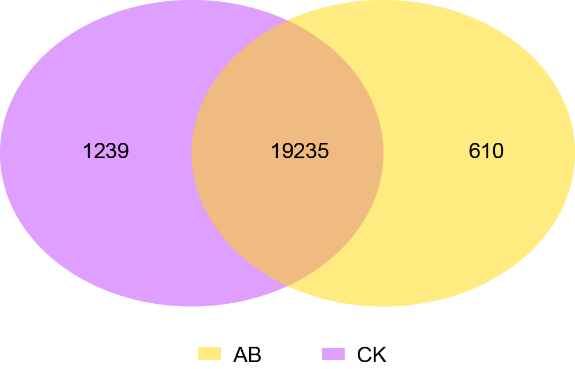


Figure S1. Venn diagram of differentially expressed genes (DEGs) in AB and CK.


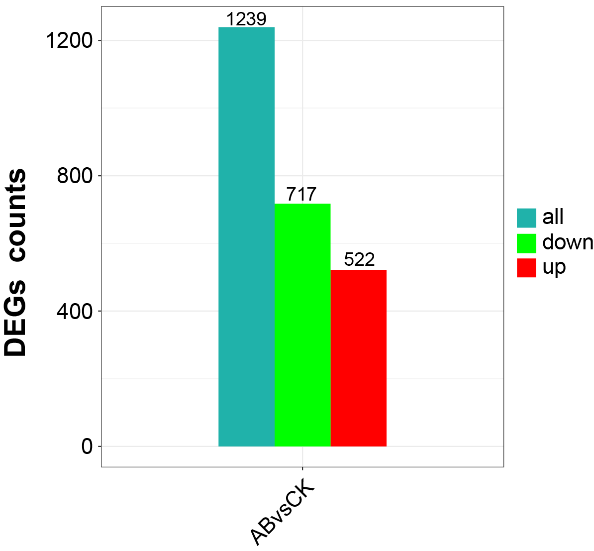


Figure S2. The counts of DEGs in AB and CK.


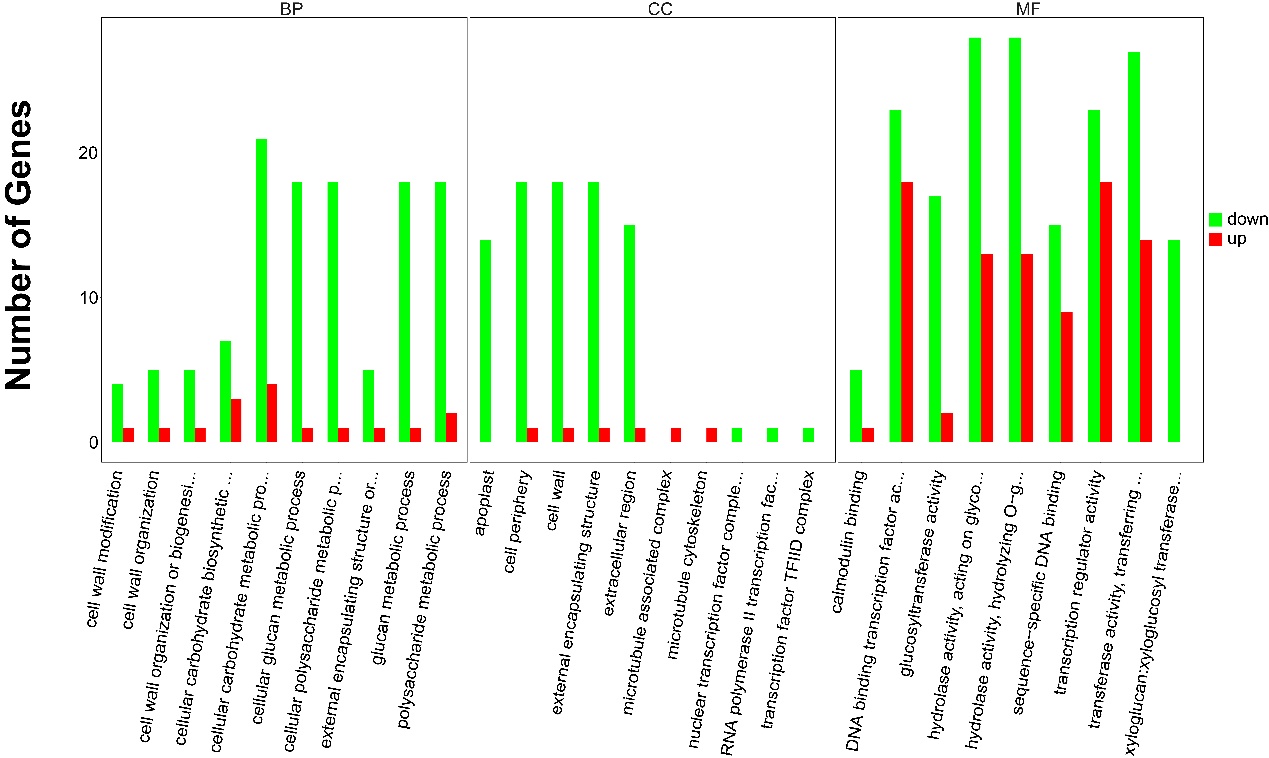


Figure S3. GO classification of DEGs between AB and CK. The X-axis represents the functional classification, and the Y-axis represents the number of genes enriched into related GO terms. BP, biological process; CC, cell component; MF, molecular function.


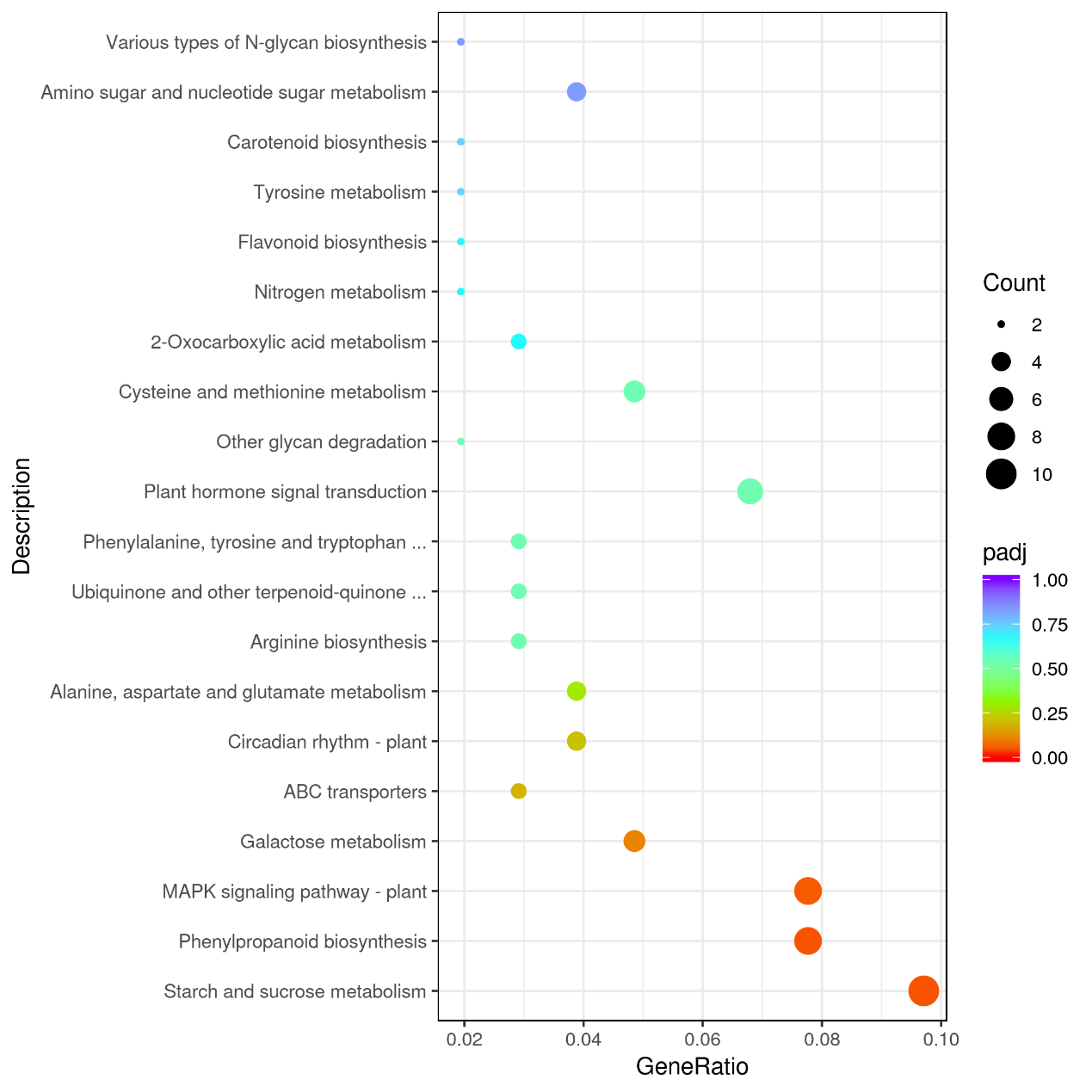


Figure S4. Top 20 enriched KEGG pathways evolving DEGs between AB and CK. The X-axis represents the gene ratio (gene ratio = number of enriched genes / number of all genes in a certain pathway), and the Y-axis represents different KEGG pathways; the size of the bubble is proportional to the number of genes enriched in the KEGG pathway; different colors represent the Q-value of enrichment.


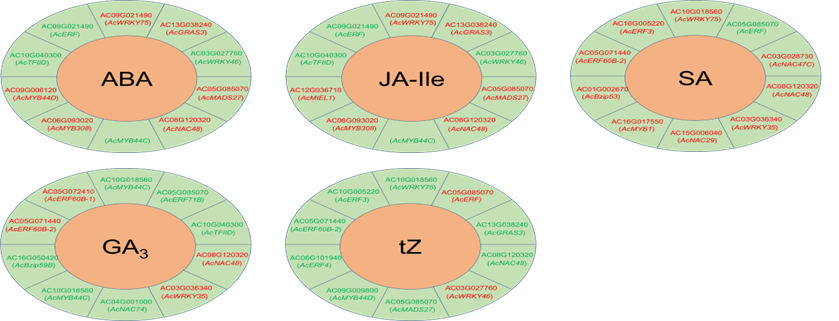


Figure S5. Top 10 correlation network of transcription factors and phytohormone contents.

The red and green fonts represent positive and negative correlations, respectively.


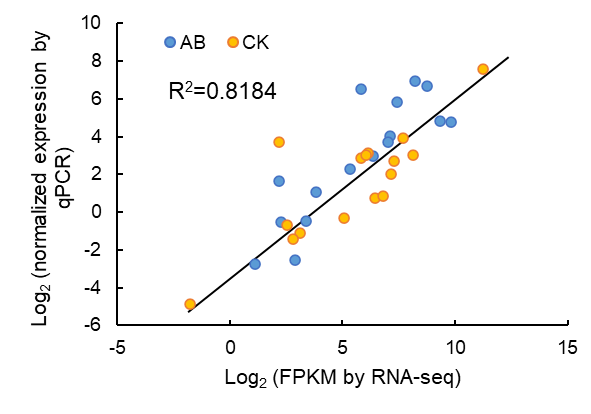


Figure S6. Correlation of gene expression results. The x-axis represents the value of Log_2_ FPKM and the y-axis represents the value of Log_2_ normalized expression level. Blue round dot represent AB. Orange round dot represent CK. R^2^ value represent the correlation between RNA-seq and qPCR results.
